# DIRECT CARE WORKER RETENTION DURING THE COVID-19 PANDEMIC AND THE IMPACT OF STATE POLICIES

**DOI:** 10.1093/geroni/igae098.0391

**Published:** 2024-12-31

**Authors:** Katherine Miller, Yang Yang, Karen Shen

**Affiliations:** Johns Hopkins University, Baltimore, Maryland, United States; Johns Hopkins University, Baltimore, Maryland, United States; Johns Hopkins University, Baltimore, Maryland, United States

## Abstract

Due to persistently low wages and poor working conditions, direct care worker shortages and high turnover existed in long-term care (LTC) industries prior to COVID-19, but became more visible during the pandemic. Some states responded to the threat of a large exodus of workers through wage-support policies (e.g., hazard pay). However, little research has examined whether these state policies were effective in mitigating the effects of the pandemic on direct care worker exits. We use the Annual Social and Economic Supplement of the Current Population Survey 2016-2022 to decompose direct care worker exits based on where they exited to: (1) unemployment or exiting the labor force, (2) a different healthcare job or industry, and (3) non-healthcare job (e.g., retail). We compare the rates of these components for direct care workers pre-pandemic (2016-2019) and during the pandemic (examining 2020 and 2021-2022 separately), and the association of these rates with state wage-support policies. Direct care worker exits were highest in 2020, with workers primarily becoming unemployed or leaving the labor force. Yet, in 2021-2022, exits decreased relative to pre-pandemic periods, primarily because fewer people switched jobs. Among those who did switch jobs, we find that workers are more likely to stay in a healthcare industry in 2021 relative to 2016-2019. We find no evidence wage support policies were associated with direct care worker retention/exits during or post-pandemic. Together, our findings suggest that broader economic trends of a shrinking workforce may be the primary driver of direct care worker exits during the pandemic.

